# Elevated PD-L1 expression predicts poor survival outcomes in patients with cervical cancer

**DOI:** 10.1186/s12935-019-0861-7

**Published:** 2019-05-23

**Authors:** Xiaobin Gu, Meilian Dong, Zheyan Liu, Yin Mi, Jing Yang, Zhigang Zhang, Ke Liu, Li Jiang, Yue Zhang, Shiliang Dong, Yonggang Shi

**Affiliations:** Department of Radiation Oncology, The First Affiliated Hospital of Zhengzhou University, Zhengzhou University, Zhengzhou, 450000 Henan People’s Republic of China

**Keywords:** Cervical cancer, PD-L1, Meta-analysis, Prognosis

## Abstract

**Background:**

Programmed cell death ligand 1 (PD-L1) expression has been shown to associate with poor prognosis in a variety of solid tumors. However, the prognostic value of PD-L1 expression in cervical cancer is still controversial. Therefore, we carried a meta-analysis to investigate the prognostic and clinicopathological impact of PD-L1 in cervical cancer.

**Methods:**

A comprehensive literature search in was performed in PubMed, Embase, Web of Science, and Cochrane Library. The correlation between PD-L1 expression and overall survival (OS), progression-free survival (PFS), and clinicopathological features was analyzed by hazard ratios (HR), odds ratios (OR) and corresponding 95% confidence intervals (CI).

**Results:**

Seven studies with 783 patients were included in this meta-analysis. The combined HR and 95% CI of OS was 2.52 (1.09–5.83), p = 0.031. The pooled results for PFS were HR = 2.07, 95% CI = 0.52–8.23, p = 0.302. The results of subgroup analysis showed that PD-L1 was a significant prognostic factor of poor OS in Asian patients (HR = 4.77, 95% CI = 3.02–7.54, p < 0.001) and of poor PFS in Asian patients (HR = 4.78, 95% CI = 1.77–12.91, p = 0.002). However, the pooled results suggested that PD-L1 was not significantly correlated with lymph node metastasis, tumor size, FIGO stage, depth of invasion, lymph-vascular invasion, or age.

**Conclusions:**

The results of this meta-analysis suggest that PD-L1 overexpression is related to poor OS in patients with cervical cancer and poor PFS in Asian patients with cervical cancer. This study also suggests that PD-L1 is a promising prognostic indicator for cervical cancer.

## Background

Cervical cancer is the fourth most common malignancy and the fourth leading cause of cancer-related deaths in females globally [1]. In 2018, it is estimated that 569,847 new cases of cervical cancer are diagnosed and 311,365 cases died from this disease worldwide [1]. With the improvement of new diagnostic techniques and therapeutic strategies, the incidence rates of cervical cancer have declined significantly in developed countries, however, the mortality is still high in developing countries. Approximately 90% of cervical cancer deaths occurred in low income and middle-income countries (LMIC) [2]. Although several prognostic biomarkers have been identified, such as lymph node status, tumor size and tumor stage, those biomarkers lack specificity and sensitivity for accurate prediction. Therefore, it is urgently needed to identify many novel and feasible prognostic markers to guide personalized treatment and predict survival outcomes of cervical cancer patients.

Programmed death-ligand 1 (PD-L1 or B7-H1) is the major ligand for programmed cell death protein 1 (PD-1). PD-L1 is expressed in immune cells, including activated T cells, B cells, dendritic cells, macrophages, and various tumor cells [3]. Normally, PD-L1 expression maintains the homeostasis of the immune response. In the healthy immune system, the activation of the PD-1/PD-L1 pathway can limit autoimmunity and inhibit the activity of T lymphocytes under an inflammatory response to infection [4]. In tumor microenvironment, cancer cells and infiltrating immune cells express PD-L1, which binds to PD-1 on T cells and then suppress the proliferative and effector responses of T cells [5, 6]. The prognostic value of PD-L1 in various have been investigated, such as breast cancer [7], non-small cell lung cancer [8], pancreatic cancer [9], renal cell carcinoma [10], and gastric cancer [11]. However, the prognostic value of PD-L1 in cervical cancer is still conflicting [12–15]. Therefore, we collected eligible data and conducted a meta-analysis to reveal the prognostic and clinical significance of PD-L1 in cervical cancer.

## Methods

### Literature search strategy

This meta-analysis was performed according to Preferred Reporting Items for Systematic Reviews and Meta-Analyses (PRISMA) Statement [16]. Relevant studies were searched from online databases PubMed, EMBASE, Web of Science, and, Cochrane Library up to Feb, 2019. The following keywords in combination with Medical Subject Headings (MeSH) terms and free words were used: “PD-L1”, “programmed cell death ligand 1”, “B7-H1”, “CD274”, “cervical carcinoma”, and “cervical cancer”. References in the retrieved articles and preceding reviews were also manually searched to identify relevant studies.

### Inclusion and exclusion criteria

Qualified studies need to meet the following inclusion criteria: [1] all patients were diagnosed as cervical cancer by pathological findings; [2] immunohistochemistry (IHC) was used to detect PD-L1 expression in tissues; [3] the relationship between PD-L1 expression and overall survival (OS) and/or progression-free survival (PFS) were provided or sufficient information was provided to estimate the hazard ratio (HR) with Tierney’s method [17]; [4] number of patients was more than 20; [5] the expression of PD-L1 was categorized into high (positive) and low (negative) groups; [6] English or Chinese articles. The exclusion criteria were as follows: [1] reviews, case reports, meeting abstracts, or letters; [2] animal studies; [3] overlapping studies.

### Data extraction and quality assessment

Two investigators (X.B., Gu and M.L., Dong) independently extracted the following information from the included studies: author, year of publication, country, number of patients, age, treatment, The International Federation of Gynecology and Obstetrics (FIGO) stage, study period, the hazard ratios (HRs) and 95% confidence intervals (CIs) for OS and PFS. Disagreements between the investigators were resolved through discussion. The quality of the selected articles was assessed according to the Newcastle–Ottawa Scale (NOS) [18]. The NOS scale consists of three factors: patient selection, comparability, and assessment of outcome. Total quality scores were ranged from 0 to 9 and studies with the final score > 6 were regarded as high-quality studies.

### Statistical analysis

The HRs and 95% CIs of each study were combined to evaluate the relationship between PD-L1 expression and the prognosis. The pooled odds ratio (OR) and 95% CI was used to assess the correlation between the PD-L1 expression and clinicopathological characteristics. Heterogeneity among studies was evaluated using Cochrane’s Q tests (Chi squared tests) and the *I*^2^ metric. Significant heterogeneity was defined as p < 0.05 for the χ^2^ test or *I*^2^ > 50% and then a random effects model was used for calculation, otherwise, a fixed effects model was applied. The potential for publication bias was assessed using the Begg’s funnel plot [19] and the Egger linear regression test [20]. All above calculations were performed using Stata version 12.0 (Stata Corporation, College Station, TX, USA). A p < 0.05 was considered as statistically significant.

## Results

### Literature selection and study characteristics

**Fig. 1:**
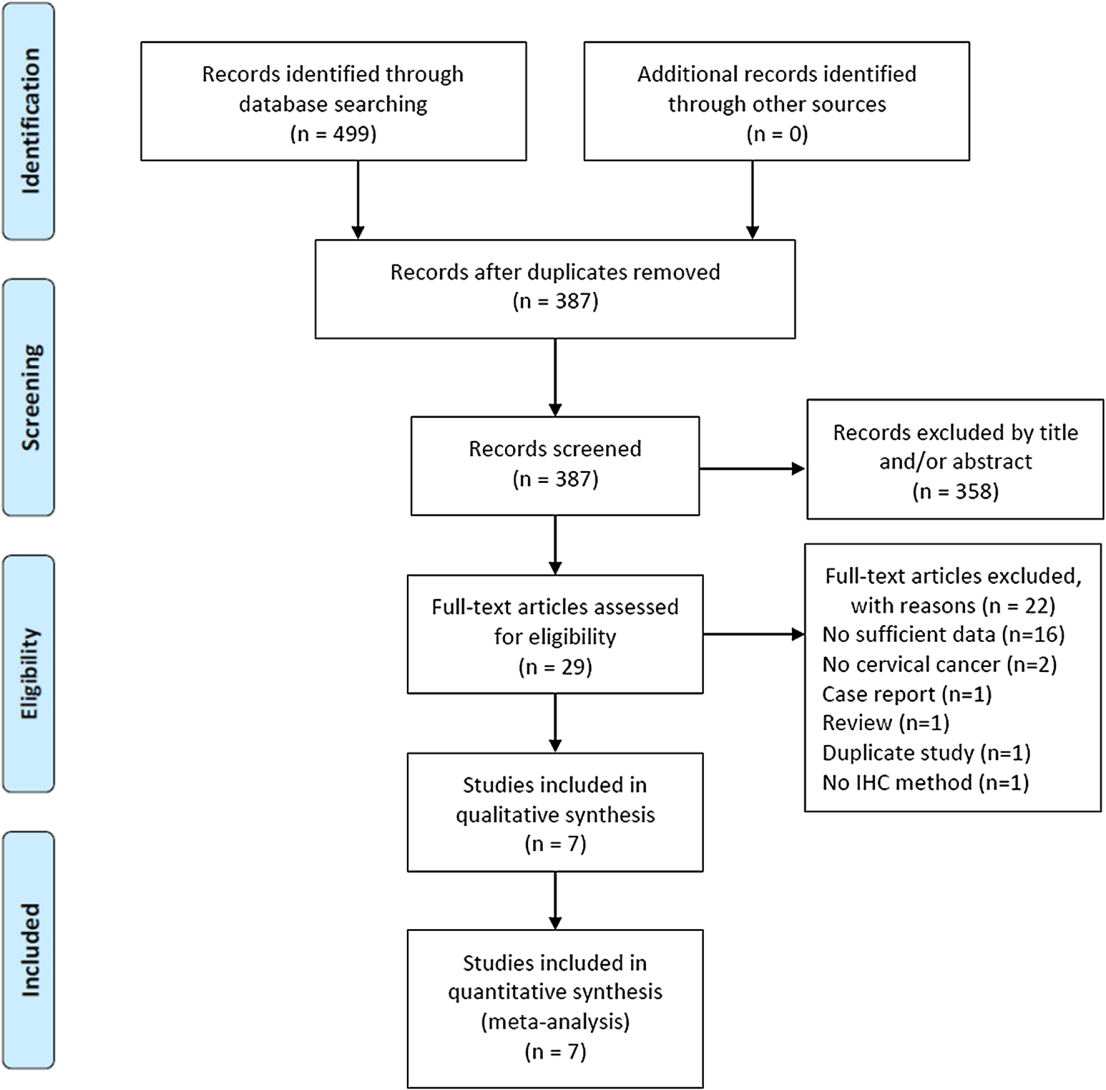
Flow chart for selection of studies

A total of 499 studies were identified through initial literature search. After duplicate records were removed, 387 studies were left. After title and/or abstracts screening, 29 articles remained for full-text assessment. By full text examination, 22 studies were excluded with various reasons. At last, 7 studies [12–15, 21–23] were included for the final meta-analysis. The detailed diagram of the above screening process is shown in Fig. 1. All 7 eligible studies were retrospective studies published between 2009 and 2018. The sample size ranged from 27 to 219 and the total sample size was 783. Three studies were conducted in China [21–23], and one in USA [12], Canada [13], Korea [14], and Japan [15], respectively. All 7 studies provided the data between PD-L1 and OS, and 3 studies [13, 14, 23] presented information for PD-L1 and PFS. Six studies [12–15, 22, 23] were published in English and one [21] was published in Chinese. The NOS scores of included studies ranged from 6 to 8, with a mean value of 7. The characteristics of the 7 eligible studies were shown in Table 1.

**Table 1:** Basic characteristics of included studies

Author	Year	Country	No. of patients	Age (year)	Treatment	FIGO stage	Detection method	Study period	Survival analysis	NOS score
Karim	2009	USA	115	46.5 (24–87)	Surgery	I–II	IHC	1985–1999	OS	7
Duan	2017	China	64	47.5 (30–65)	Surgery	I–IIA	IHC	2013–2015	OS	6
Enwere	2017	Canada	120	44 (39–49)	CCRT	IB–IVA	IHC	1999–2008	OS, PFS	7
Kim	2017	Korea	27	46 (34–71)	Surgery	IB1–IIA	IHC	2011–2012	OS, PFS	8
Feng	2018	China	219	49 (26–75)	Mixed	I–IV	IHC	2013–2016	OS	7
Kawachi	2018	Japan	148	45 (30–72)	Surgery	I–II	IHC	2001–2014	OS	7
Wang	2018	China	90	46 (23–71)	Surgery	IB1–IIA2	IHC	2009–2012	OS, PFS	7

CCRT, concurrent chemo-radiotherapy; IHC, immunohistochemistry; OS, overall survival; PFS, progression-free survival; NOS, Newcastle–Ottawa scale

### Association between PD-L1 expression and OS, PFS

**Fig. 2:**
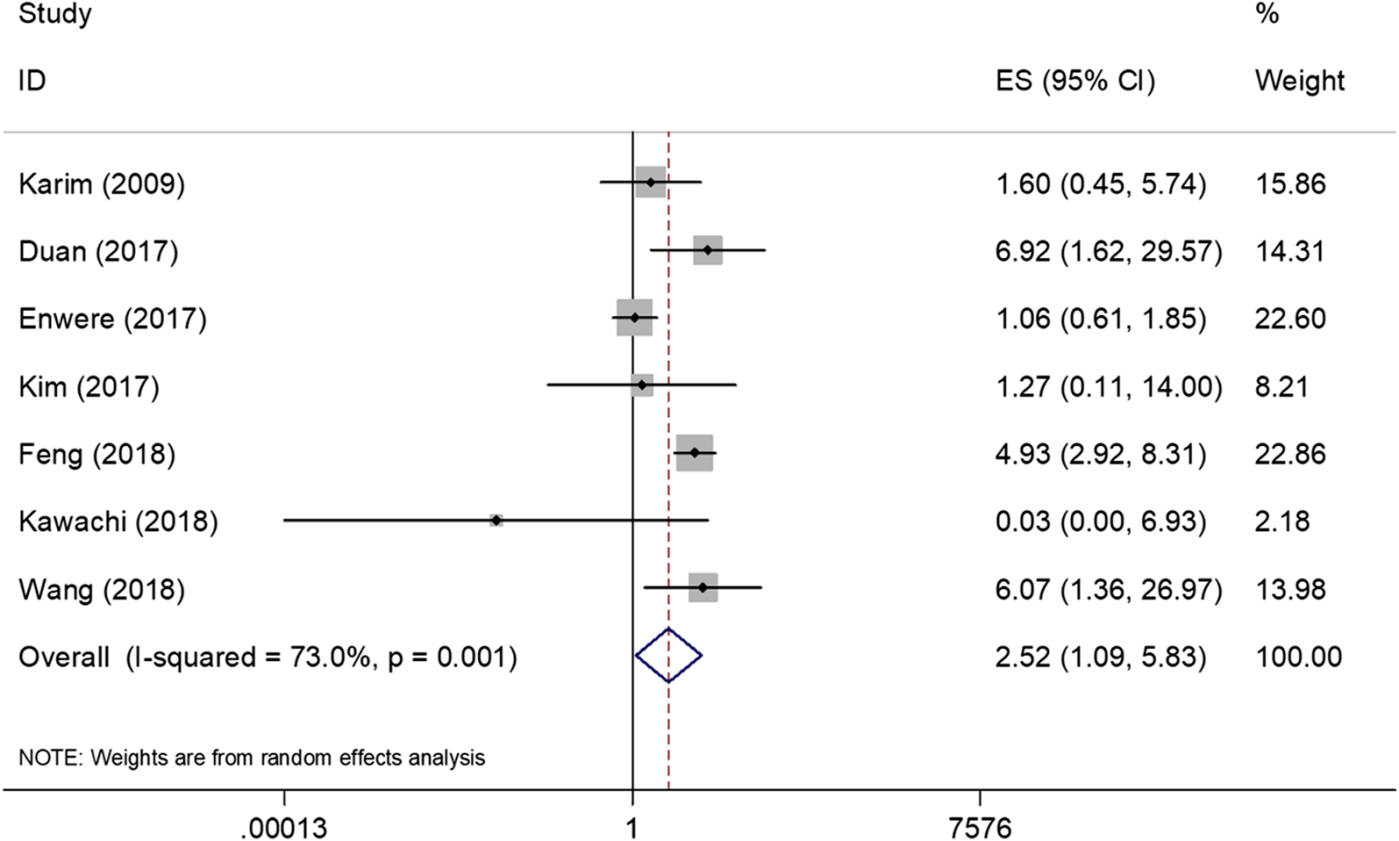
Forest plot showing pooled hazard ratio for OS and PD-L1 expression in cervical cancer

**Fig. 3:**
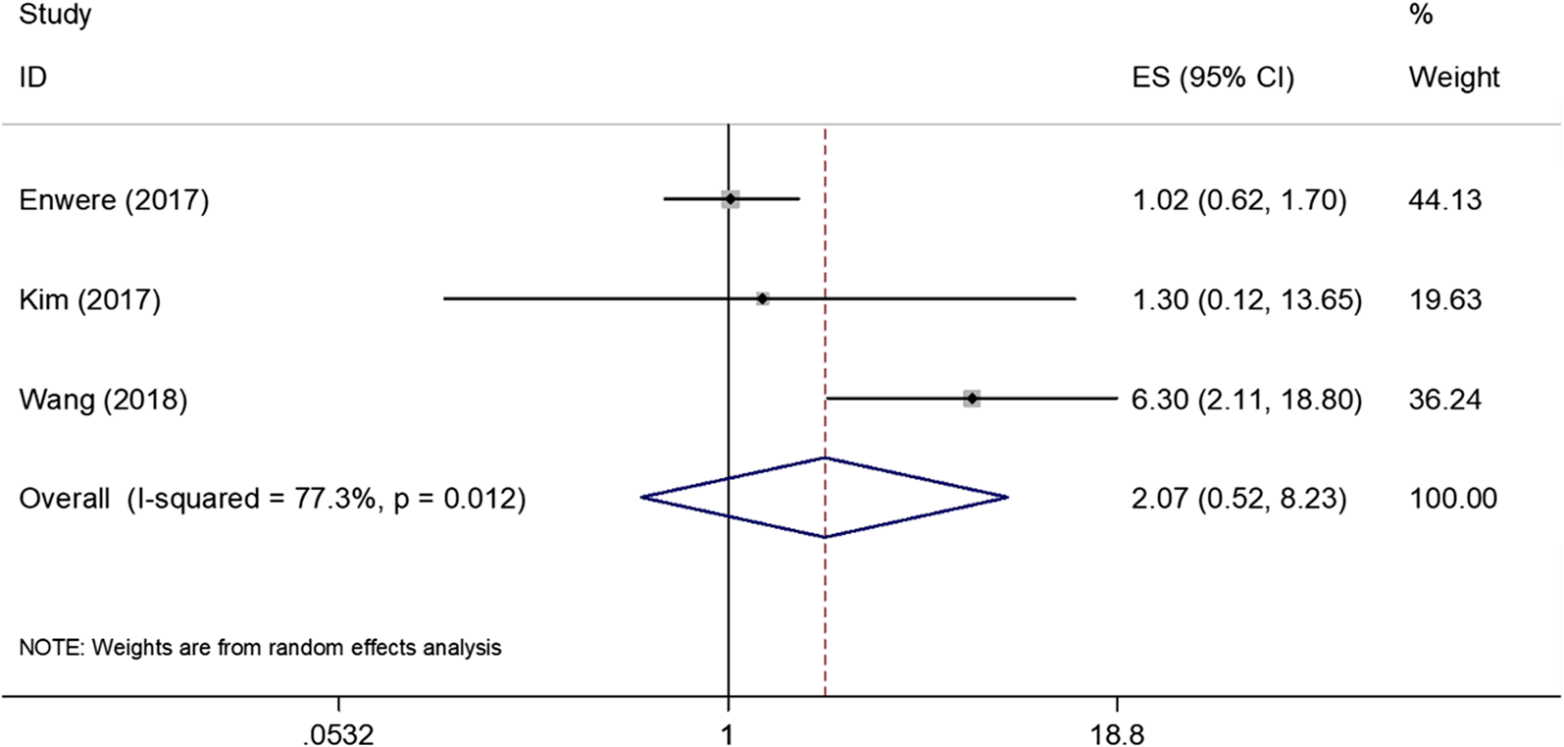
Forest plot showing pooled hazard ratio for PFS and PD-L1 expression in cervical cancer

All 7 included studies [12–15, 21–23] presented the correlations between PD-L1 and OS. The pooled HR and 95% CI of OS was 2.52 (1.09–5.83), p = 0.031. The heterogeneity was not significant (*I*^2^ = 73%, p = 0.001) therefore the random-effects model was used (Fig. 2). Three studies [13, 14, 23] reported the associations between PD-L1 and PFS. As shown in Fig. 3, the results were HR = 2.07, 95% CI = 0.52–8.23, p = 0.302, the random-effects model was applied because of significant heterogeneity (*I*^2^ = 77.2%, p = 0.012).

### Subgroup analysis

To further investigate the prognostic value of PD-L1 in different subpopulations, the subgroup analysis was conducted. The subgroup analysis was performed based on the following factors: ethnicity (Asian or Caucasian), sample size (< 100 or ≥ 100), and treatment (surgery or non-surgery). The results showed that high expression of PD-L1 was a significant prognostic factor of poor OS in Asian patients (HR = 4.77, 95% CI = 3.02–7.54, p < 0.001), but not in Caucasian patients (HR = 1.13, 95% CI = 0.68–1.88, p = 0.634) (Table 2). In addition, PD-L1 was also a significant marker of OS in studies with sample < 100 (HR = 5.01, 95% CI = 1.93–13.03, p = 0.001) and patients receiving surgery (HR = 3.04, 95% CI = 1.42–6.48, p = 0.004). However, PD-L1 was not significantly associated with poor OS in studies with sample ≥ 100 or patients receiving non-surgery treatment. As for PFS, the subgroup analysis indicated that PD-L1 showed significant prognostic value in Asian patients (HR = 4.78, 95% CI = 1.77–12.91, p = 0.002) (Table 2).

**Table 2:** Subgroup analysis of PD-L1 and OS, PFS in cervical cancer

Factors	No. of studies	Effects model	HR (95% CI)	p	Heterogeneity *I*^2^(%) Ph	
OS						
Total	7	Random	2.52 (1.09–5.83)	0.031	73	0.001
Ethnicity						
Asian	5	Fixed	4.77 (3.02–7.54)	< 0.001	18	0.3
Caucasian	2	Fixed	1.13 (0.68–1.88)	0.634	0	0.562
Sample size						
< 100	3	Fixed	5.01 (1.93–13.03)	0.001	0	0.471
≥ 100	4	Random	1.75 (0.55–5.59)	0.344	83.7	< 0.001
Treatment						
Surgery	5	Fixed	3.04 (1.42–6.48)	0.004	36.6	0.177
Non-surgery	2	Random	2.29 (0.51–10.34)	0.28	93.6	< 0.001
PFS						
Total	3	Random	2.07 (0.52–8.23)	0.302	77.3	0.012
Ethnicity						
Asian	2	Fixed	4.78 (1.77–12.91)	0.002	28.7	0.236
Caucasian	1	NA	1.02 (0.62–1.69)	0.939	NA	NA

OS, overall survival; PFS, progression-free survival; NA, not available

### Correlation of PD-L1 expression with clinicopathological characteristics

**Fig. 4:**
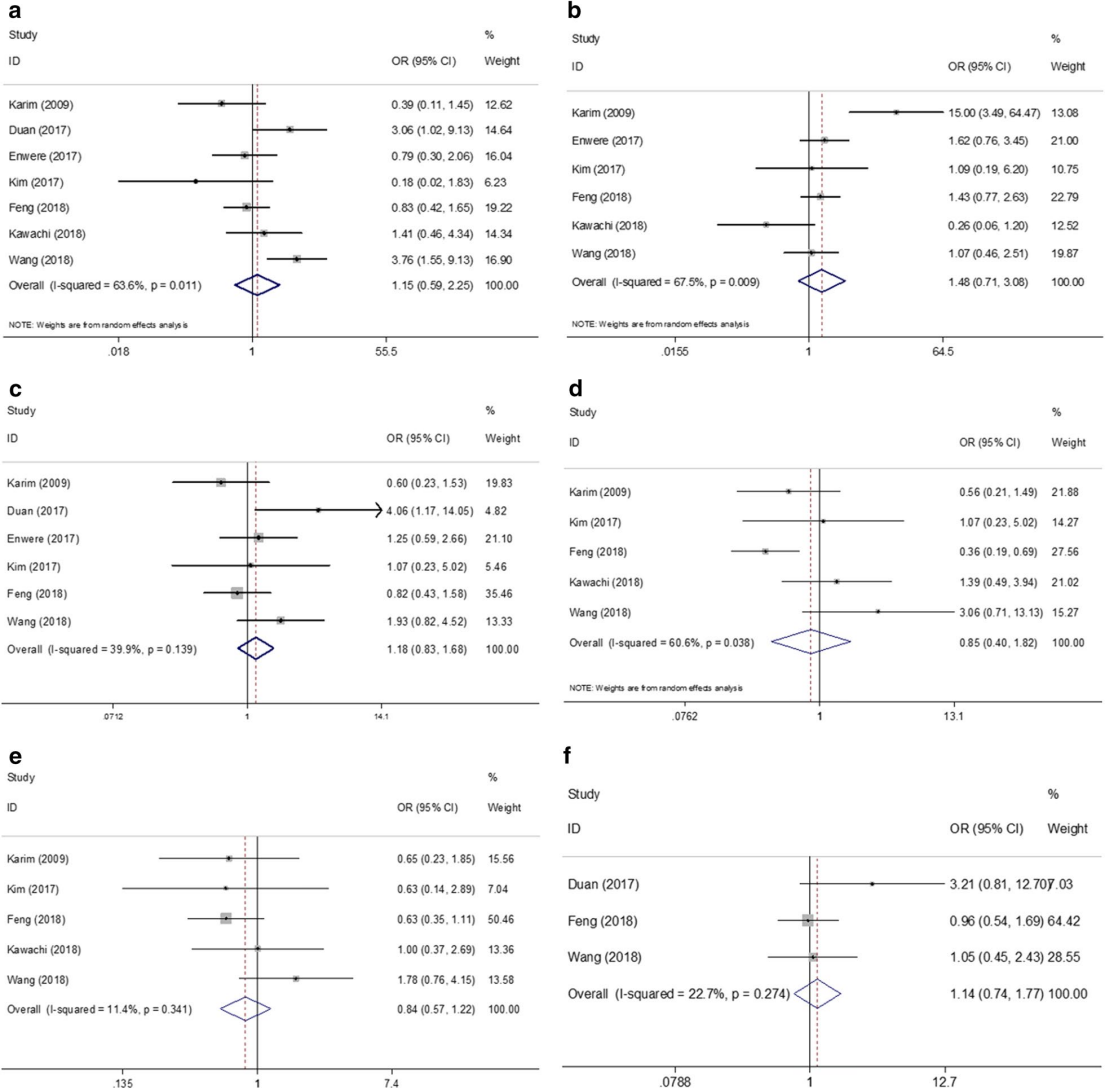
Meta-analysis of the association between PD-L1 and clinicopathological factors in cervical cancer: **a** lymph node metastasis (yes vs no), **b** tumor size (mm) (≥ 40 vs < 40), **c** FIGO stage (II vs I), **d** depth of invasion (mm) (≥ 10 vs < 10), **e** lymph-vascular invasion (yes vs no), **f** age (y) (≥ 45 vs < 45)

To identify the impact of PD-L1 on clinicopathological characteristics of cervical cancer, we investigated the association between PD-L1 overexpression with six factors. As shown in Table 3 and Fig. 4, the pooled results suggested that PD-L1 was not significantly correlated with lymph node metastasis (n = 7, OR = 1.15, 95% CI = 0.59–2.25, p = 0.682), tumor size (n = 6, OR = 1.48, 95% CI = 0.71–3.08, p = 0.294), FIGO stage (n = 6, OR = 1.18, 95% CI = 0.83–1.68, p = 0.345), depth of invasion (n = 5, OR = 0.85, 95% CI = 0.4–1.82, p = 0.674), lymph-vascular invasion (n = 5, OR = 0.84, 95% CI = 0.57–1.22, p = 0.357), or age (n = 3, OR = 1.14, 95% CI = 0.74–1.77, p = 0.554).

**Table 3:** Associations between PD-L1 and clinical factors in cervical cancer

Clinical factors	No. of studies	Effects model	OR (95% CI)	p	Heterogeneity *I*^2^ (%) Ph		Begg’s p	Egger’s p
Lymph node metastasis (yes vs no)	7	Random	1.15 (0.59–2.25)	0.682	63.6	0.011	0.368	0.543
Tumor size (mm) (≥ 40 vs < 40)	6	Random	1.48 (0.71–3.08)	0.294	67.5	0.009	0.707	0.93
FIGO stage (II vs I)	6	Fixed	1.18 (0.83–1.68)	0.345	39.9	0.139	0.707	0.85
Depth of invasion (mm) (≥ 10 vs < 10)	5	Random	0.85 (0.4–1.82)	0.674	60.6	0.038	0.221	0.062
Lymph-vascular invasion (yes vs no)	5	Fixed	0.84 (0.57–1.22)	0.357	11.4	0.341	1	0.758
Age (y) (≥ 45 vs < 45)	3	Fixed	1.14 (0.74–1.77)	0.554	22.7	0.274	0.296	0.24

FIGO, The International Federation of Gynecology and Obstetrics

### Publication bias

**Fig. 5:**
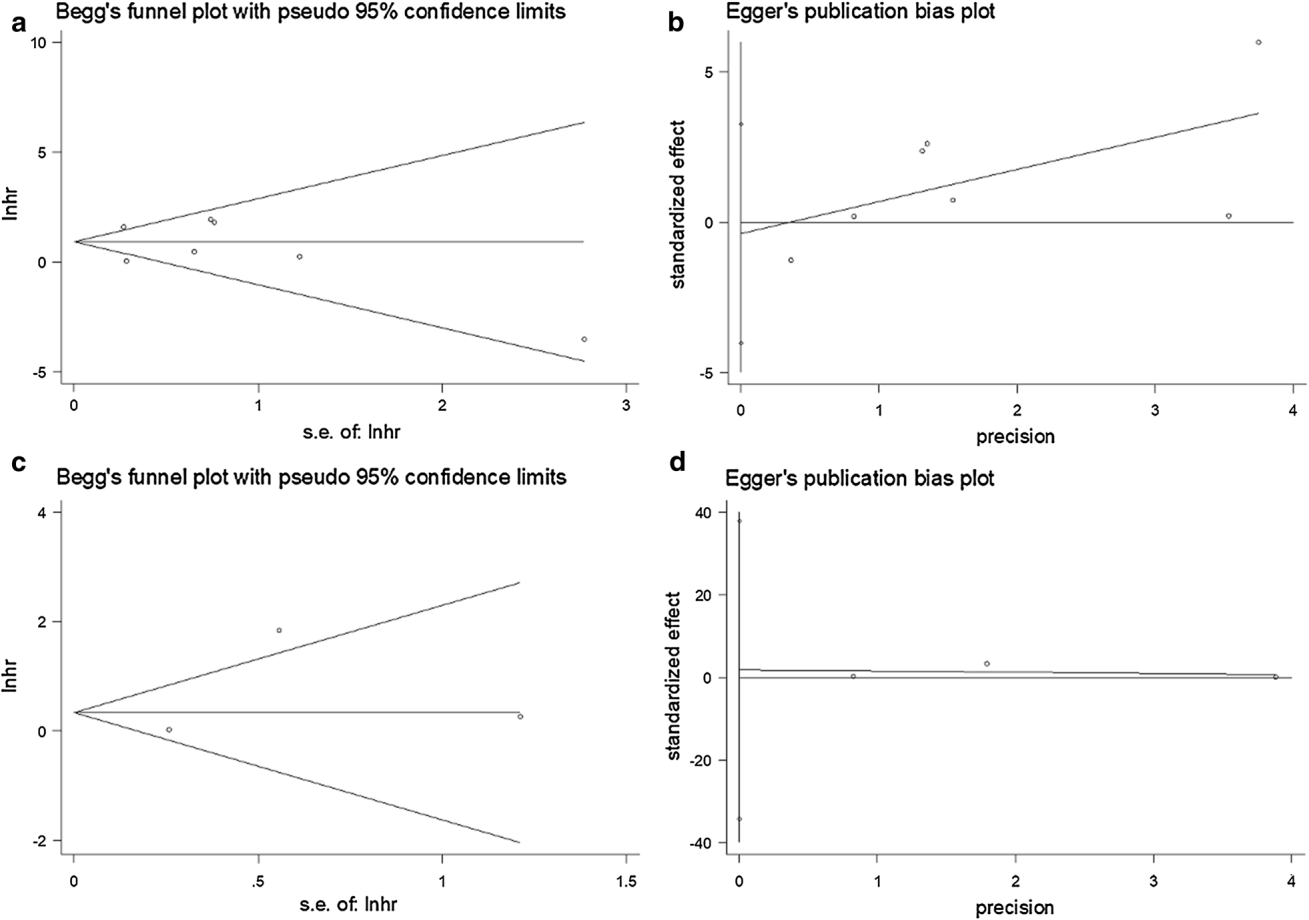
Publication bias. **a** Begg’s test for OS (p = 0.548), **b** Egger’s test for OS (p = 0.798), **c** Begg’s test for PFS (p = 1), **d** Egger’s test for PFS (p = 0.638)

Begg’s funnel plots and Egger’s linear regression test were used to evaluate the publication bias of the eligible studies. The results showed that no significant publication bias was detected for OS (Begg’s p = 0.548, Egger’s p = 0.798, Fig. 5) or PFS (Begg’s p = 1, Egger’s p = 0.638, Fig. 5). The results of publication bias for PD-L1 and clinicopathological characteristics were listed in Table 3. All p-values of publication bias were > 0.05, indicating the credibility of this meta-analysis.

## Discussion

The present meta-analysis is the first to evaluate the association between PD-L1 overexpression and survival in patients with cervical cancer. In this study, we pooled the data from 7 eligible studies with 783 patients and found that PD-L1 was a significant prognostic factor for poor OS, but not for PFS. Further subgroup analysis revealed that PD-L1 overexpression had enhanced prognostic function of poor OS in Asian patients, moreover, PD-L1 also indicated poor PFS in Asian patients. Nevertheless, the correlation of PD-L1 and several clinicopathological features were not statistically significant, which might imply the clinical roles of PD-L1 in cervical cancer diagnosis.

PD-L1 is an important immune regulatory molecule that was reported to be critically involved in the immune escape mechanism of various cancer cells [24]. In many solid tumors, the overexpression of PD-L1 can lead to immunosuppressive tumor microenvironment and prevent cell-mediated lysis. In addition, the expression of PD-1 in tumor infiltrating lymphocytes is another key point of immune escape mediated by PD-1/PD-L1 [25]. Interaction of PD-1/PD-L1 results in blocking T cell activation and inhibiting TCR signal transduction and CD28-CD80 co-stimulation [26]. PD-L1 is also expressed in activated immune cells including dendritic cells, macrophages, B cells, T cells and natural killer cells [27].

Previous studies also investigated the prognostic role of PD-L1 in various solid tumors. A recent study [28] demonstrated that expression level of PD-L1 was associated with the OS in gastric cancer (HR = 1.46, 95% CI = 1.08–1.98, p = 0.01). A previous work conducted by Ni et al. also showed that the pooled HR of (1.34, 95% CI 1.02–1.65, p = 0.01) indicated the association of PD-L1 expression with OS in colorectal cancer patients [29]. Additionally, Ni’s study also reported the expression of PD-L1 was positively correlated with the lymph node metastasis in colorectal cancer [29]. The prognostic value was also presented in other solid malignancies including prostate cancer [30], esophageal squamous cell carcinoma [31], glioma [32], Osteosarcoma [33], and non-small cell lung cancer [34]. The results of the present meta-analysis were in line with the results of other types of cancer. The present study is the first meta-analysis on PD-L1 and cervical cancer to date. Previous meta-analyses [35, 36] on PD-L1 and survival in solid tumors only included one study [12] of cervical cancer, the results provided limited information on the prognostic value of PD-L1 in cervical cancer. Therefore, our meta-analysis was comprehensive and convincing. A recent study [37] systematically reviewed the present and ongoing clinical researches on PD-1/PD-L1 inhibitors in cervical cancer. The patients showed favorable objective response rate (ORR) and disease control with PD-1/PD-L1 inhibitors treatment. We highlight the importance of survival as the primary evaluation endpoint in clinical trials on PD-1/PD-L1 inhibitors in cervical cancer. Furthermore, in the present study, our results that PD-L1 overexpression predicts worse OS might provide implications for future clinical design and assessment.

There are some limitations to this study that should be acknowledged. First, the sample size was relatively small. Although 7 studies were included for analysis, the total sample size was 783, the limited amount may compromise the generality of the results. Second, only articles published in English and Chinese were included in this meta-analysis. Because of our restriction of availability to articles published in other languages, the studies from other countries may be neglected. Third, there may be inconsistent data in the included studies, as they used different cutoff values for identifying PD-L1 overexpression.

## Conclusions

This meta-analysis demonstrated that PD-L1 overexpression is related to poor OS in patients with cervical cancer and poor PFS in Asian patients with cervical cancer. PD-L1 overexpression had non-significant association with clinical characteristics in cervical cancer. This study also suggests that PD-L1 is a promising prognostic indicator for cervical cancer. Due to abovementioned limitations, further large-scale prospective studies are needed to warrant the results.

## Data Availability

Please contact author for data requests.

## Abbreviations

PD-L1: programmed cell death ligand 1
OS: overall survival
PFS: progression-free survival
HR: hazard ratio
OR: odds ratios
CI: confidence interval
LMIC: low income and middle-income countries
PRISMA: Preferred Reporting Items for Systematic Reviews and Meta-Analyses
MeSH: Medical Subject Headings
IHC: immunohistochemistry
FIGO: The International Federation of Gynecology and Obstetrics
